# Soft Biomimetic Approach for the Development of Calcinosis-Resistant Glutaraldehyde-Fixed Biomaterials for Cardiovascular Surgery

**DOI:** 10.3390/biomimetics8040357

**Published:** 2023-08-10

**Authors:** Alyona I. Zvyagina, Vladislav V. Minaychev, Margarita I. Kobyakova, Yana V. Lomovskaya, Anatoliy S. Senotov, Kira V. Pyatina, Vladimir S. Akatov, Roman S. Fadeev, Irina S. Fadeeva

**Affiliations:** 1 Institute of Theoretical and Experimental Biophysics, Russian Academy of Sciences, 142290 Pushchino, Russiaakatov.vladimir@gmail.com (V.S.A.); fadeevrs@gmail.com (R.S.F.); 2 Pushchino State Institute of Natural Science, 142290 Pushchino, Russia

**Keywords:** structural valve degeneration, matrix damage, glutaraldehyde-fixed biomaterials, pericardium, immunogenicity, calcification, calcinosis-resistant biomaterials, heart valve bioprosthesis

## Abstract

Pathological aseptic calcification is the most common form of structural valvular degeneration (SVD), leading to premature failure of heart valve bioprostheses (BHVs). The processing methods used to obtain GA-fixed pericardium-based biomaterials determine the hemodynamic characteristics and durability of BHVs. This article presents a comparative study of the effects of several processing methods on the degree of damage to the ECM of GA-fixed pericardium-based biomaterials as well as on their biostability, biocompatibility, and resistance to calcification. Based on the assumption that preservation of the native ECM structure will enable the creation of calcinosis-resistant materials, this study provides a soft biomimetic approach for the manufacture of GA-fixed biomaterials using gentle decellularization and washing methods. It has been shown that the use of soft methods for preimplantation processing of materials, ensuring maximum preservation of the intactness of the pericardial ECM, radically increases the resistance of biomaterials to calcification. These obtained data are of interest for the development of new calcinosis-resistant biomaterials for the manufacture of BHVs.

## 1. Introduction

Heart valve defects are the main cause of disability and deterioration of quality of life and are the leading cause of cardiovascular morbidity and mortality worldwide [1]. Considering the general increase in life expectancy of the population, as well as the growth of predisposing factors for infectious endocarditis, it is predicted that the prevalence of valvular heart diseases will only increase in the coming decades [2,3].

Currently, the standard therapy for valvular diseases is the surgical replacement of dysfunctional native valves with artificial prostheses such as mechanical or bioprosthetic heart valves (BHV) [4]. Over the past two decades, the use of BHVs in the treatment of valvular heart diseases has become dominant owing to the development of transcatheter heart valve replacement (TAVR) technology [5,6]. In addition, unlike mechanical prostheses, BHVs have several advantages: no need for anticoagulant therapy and suitable hemodynamic properties similar to native heart valves [7,8]. However, the main disadvantage limiting the use of BHVs, especially for young patients, is inevitable structural valve degeneration (SVD) [6,7,8]. SVD is defined as an irreversible internal change in the valve leading to aseptic calcification, deposition of pannus, and rupture of the valves, which ultimately requires repeated surgical intervention with a high risk for the patient [6,9].

Aseptic calcification is the most common pathological form of SVD [10] and is considered the endpoint of BHV dysfunction, leading to progressive stiffness, fragility, and valve obstruction [9]. Previously, calcification of BHVs was considered a passive degenerative process; however, it is now considered a complex mechanism actively regulated by several factors [6]. Recent studies have shown that several factors are involved in the development of BHV calcification, including preimplantation processing of materials, composition of biomaterials, and immune response of the recipient’s body [4].

Over the past decade, an increasing amount of data has accumulated, convincingly indicating the decisive involvement of the immune response in the calcification and degradation of BHV [11,12,13]. Studies of calcified BHV tissues have shown significantly increased infiltration of immune cells, including T cells, macrophages, B cells, and neutrophils, accompanied by an increased concentration of inflammatory cytokines and antibody levels in the fabric [12,13,14]. Several reasons for the development of such immune reactions are assumed: (1) nonspecific protein adsorption [15,16], which leads to the activation of the complement system and platelets, a cascade of coagulation and recruitment of immune cells; (2) the presence in the biomaterial of unmasked (unfixed) xenoantigens of a carbohydrate nature (α-Gal, Neu5Gc, Sda) [4,6,17]. In addition, BHVs are usually made from ECM-based biomaterials obtained from tissues of xenogenic origin (bovine or porcine pericardium) using specific treatments, including decellularization and fixation of tissues in glutaraldehyde (GA). Some researchers believe that such preimplantation treatment, namely the agents used (residual detergents, GA, etc.), may also be one of the reasons for the development of the recipient’s inflammatory immune response to the implanted material [18,19,20,21] and the development of BHV calcification.

It is also assumed that preservation of the native ECM structure during processing is highly desirable to reduce the recipient’s immune response to decellularized ECM biomaterials [22,23,24]. GA-fixed tissues are not completely resistant to enzymatic degradation and are not metabolically inert [4]. Thus, the destruction of the ECM of biomaterials during preimplantation treatment and their enzymatic degradation during the body’s immune response after implantation contribute to exposing hole zones for the nucleation of hydroxypaptite crystals on the BHV collagen matrix, which provides spatial relief of calcification. However, owing to a lack of effective methods for assessing structural damage to the ECM, the destructive effects of processing methods and the effect of related structural damage on the biological properties of materials are often discussed [21,25,26,27]. Data on the effect of damage to the structure of GA-fixed biomaterials on the biocompatibility and calcinosis resistance of BHV are scarce or completely absent.

This article presents a comparative study of the effects of several processing methods on the degree of preimplantation damage of ECM-based GA-fixed biomaterials, as well as on the degree of their biocompatibility in vitro and aseptic calcification in vivo. Based on the assumption that the preservation of structural integrity during ECM processing will allow the creation of calcinosis-resistant materials, this study presents the authors’ method of manufacturing GA-fixed ECM-based biomaterials using gentle methods of decellularization and chemical stabilization.

## 2. Materials and Methods

### 2.1. Reagents

MycoFluor™ mycoplasma detection kits were purchased from Thermo Scientific (Waltham, MA, USA). The fetal bovine serum was from Gibco (Gibco, Sigma Aldrich Company Ltd., Waltham, MA, USA). Sodium dodecyl sulfate, Sodium Deoxycholate Monohydrate, Triton™ X-100 solution, Glutaraldehyde and Formaldehyde solution, Isopropyl and Ethyl Alcohol, Accutase cocktail, fluorescent dyes Calcein AM, Propidium iodide and Bisbenzimide Hoechst 33342, Culture media alfa-MEM and DMEM, L-glutamine, gentamicin sulfate, Alcian Blue 8GX, Biebrich scarlet, Fast Green FCF, Schiff’s reagent and other chemicals were purchased from Sigma-Aldrich (St. Louis, MO, USA or Gibco, Sigma-Aldrich Company Ltd., Waltham, MA, USA). Dioxidine^®^ (Hydroxymethylquinoxalindioxide, 10 mg/mL) was purchased from JSC “Valenta Pharm” (Moscow, Russia). Mayer’s hematoxylin, eosin H, Alizarin red S, toluidine blue, neutral buffered formalin, and other reagents for histological analysis were purchased from Labiko LLC (Saint Petersburg, Russia). All other reagents used were of analytical reagent quality.

### 2.2. Preparation of GA-Fixed Biomaterials

The freshly harvested pericardium of mature bulls was transported to the laboratory in a cold saline solution (0.9% NaCl, 12 ± 2 °C). Next, the pericardium was placed in 0.02% EDTA solution for 2 h with constant stirring on a shaker (40 rpm) at room temperature with a tissue-to-volume ratio of 1:50. The tissue was then mechanically cleaned of adipose tissue and treated as described in Table 1.

Reagents used by leading BHV manufacturers were used for the preimplantation treatment. The damaging effect of SDS on the ECM is well known [28,29,30]. At the same time, the results of comparative studies showed that SDC is less aggressive than SDS and damages collagen fibrils to a lesser extent [31]. In addition, alcohols in high concentrations can cause critical dehydration of the matrix, leading to the loss of the physical properties of its fibers and, as a rule, irreversible changes in their structure [32]. In addition, it has been shown that the use of GA not only increases the cytotoxicity of biomaterials based on ECM but also leads to aggregation and conformational changes of collagen molecules and can contribute to a decrease in the protective hydration of tissues [33,34]. The damaging effect of storage solutions on the structure of ECM is not covered in the literature; therefore, the contribution of this stage of processing to the structural changes in GA-fixed biomaterials was evaluated during the study. As a result, all studied materials were divided into the following groups: A—aggressive processing methods (groups A1 and A2); M—processing, presumably having a moderate (medium) effect on ECM (groups M1 and M2); and S—soft processing method (group S), developed by the authors (patent RU2678966C1).

### 2.3. ECM Damage Assessment

To assess the degree of damage to ECM materials after processing, native pericardium (group N) was used as a comparative control. The degree of damage to the collagen ECM was assessed by analyzing the histological preparations stained with Lillie’s trichrome. The number of damaged collagen fibers was calculated in the field of vision (100 × 100 µm, *n* = 12) and expressed as a percentage of the total number of collagen fibers analyzed. A qualitative assessment of the degree of ECM damage was also performed based on the intensity of fuchsinophilic saturation of histological preparations stained with Alcyan blue-PAS.

The morphological characteristics of all samples before and after the preimplantation treatment were studied using a Tescan VEGA III (scanning electron microscopy (SEM), Brno, Czech Republic) equipped with energy dispersive spectroscopy (EDS; INCA Energy Oxford Instruments, Abingdon, UK). The surface of the lyophilized samples (*p.serosum*) was covered with gold by Q150R Quorum Technologies (Lewes, UK) at a pressure of 7.3 × 10^−2^ Pa in the column and 1.5 × 10^−1^ Pa in the chamber. The area of damage was calculated in the field of vision (400 × 400 μm, *n* = 12) using the non-commercial ImageJ software (https://imagej.nih.gov (accessed on 16 March 2023)) and was expressed as a percentage of the total area of the estimated surface.

### 2.4. Cell Cultures

Human adipose tissue-derived mesenchymal stem cells (hMSC-AT) were obtained from the CLS Cell Lines Service GmbH (Eppelheim, Deutschland). These cells were cultured in alfa-MEM medium (Sigma-Aldrich, St. Louis, MO, USA) supplemented with 10% fetal bovine serum (Gibco, Sigma-Aldrich Company Ltd., Waltham, MA, USA) and 40 µg/mL of gentamicin sulfate (Sigma-Aldrich, St. Louis, MO, USA) at 37 °C in a humidified atmosphere of 5% CO_2_. Testing of cell cultures for mycoplasma infection was performed using the MycoFluor™ mycoplasma detection kit. Infection of cell cultures with mycoplasma was not detected.

### 2.5. Cytotoxicity Assay

A cytotoxicity assay was carried out to study the contact toxicity (cultivation on the surface of samples) and non-contact toxicity (cultivation with extracts from samples) of the experimental materials.

Before seeding the cells, the materials were subjected to standard washing in saline solution for 2 h, in accordance with the deadlines for washing chemically stabilized bioprostheses of heart valves, according to the manufacturer’s recommendations (standard from 3 min to 2 h). To determine the potential for re-endothelialization of the experimental materials, additional washing of samples was carried out in a nutrient medium of DMEM with 10% FBS for 4 days with a tissue-to-volume ratio of 1:30, simulating the leaching of stabilizing agents and capping of free aldehyde groups with blood plasma amino acids observed in the recipients after implantation of BHV into the systemic circulation. 

Extracts from the materials (non-contact toxicity assay) were carried out in an alfa-MEM medium with 10% FBS at 37 °C in a humidified atmosphere of 5% CO_2_ for 72 h, with constant stirring on a shaker (40 rpm). The media samples were filtered through a sterile membrane with a pore diameter of 0.22 µm (Millex-GP Filter, Millipore, Burlington, MA, USA) and used in experiments.

The calculation of living and dead cells during cultivation on the surface (contact cytotoxicity) of the experimental materials was performed 96 h after seeding. The cells were detached from the surface of the samples (*p.serosum*) using an Accutase cocktail (Sigma-Aldrich, St. Louis, MO, USA).

To determine non-contact cytotoxicity, cells were seeded into the wells of a 96-well plate, and 200 µL of extract was added. The calculation of living and dead cells was performed 96 h after seeding and the addition of the extracts.

In the case of contact and non-contact toxicity, cells were stained with calcein AM (200 nM) and propidium iodide (1 µg/mL) for 25 min at 37 °C. Live and dead cells were analyzed using an Accuri C6 flow cytometer (BD Bioscience, Franklin Lakes, NJ, USA).

### 2.6. Cell Proliferation Assay

The number of cells in suspension after their detachment with the Accutase cocktail was analyzed using a BD Accuri C6 flow cytometer (BD Bioscience, Franklin Lakes, NJ, USA). The mitotic cells were assessed by means of fluorescence of cells stained with nuclear dye H33342 at a concentration of 1 µg/mL and counting the number of mitotic cells using a DM 6000 fluorescence microscope (Leica, Wetzlar, Germany) [35]. The total number of analyzed cells in randomly selected fields was at least 500. 

### 2.7. Animals

Sixty male Wistar rats weighing 180–200 g (age two months) were used. The animals were individually housed in a temperature-controlled room (22 °C) and fed a standard diet with full access to water and food. The experiments were performed according to the Regulations for Studies with Experimental Animals (Decree of the Russian Ministry of Health on 12 August 1997, No. 755). The protocol was approved by the Commission on Biological Safety and Ethics at the Institute of Theoretical and Experimental Biophysics, Russian Academy of Sciences (5 March 2022; protocol N27/2022). For the experiments, the rats were divided into six groups (five in each group), and independent replicates were performed for each group. 

The generally accepted model of ectopic (subcutaneous) implantation of biomaterials was used (ISO 10993-6 “Biological evaluation of medical devices—Part 6: Tests for local effects after implantation”. The surgeries were performed under general anesthesia with xylazine (13 mg/kg; Interchemie, Netherlands) and zoletil 7 mg/kg (Virbac, Carros, France). To implant the specimens, a 1.5-cm-wide transverse skin incision was made in the dorsal interscapular area, subcutaneous pockets were formed parallel to the skin using a smooth trocar, and the specimens were implanted at a depth of at least 2 cm from the incision line. The samples were implanted with full interstitial contact without restriction chambers or meshes. All the experimental samples (groups A1, A2, M1, M2, and S) were used for implantation. The samples (leaflets) of PERIMOUNT RSR bioprosthesis (Edwards Lifesciences, Irvine, CA, USA) were used as a positive control (Edw group). 

For postsurgical recovery, the animals were exposed to a heating plate until awakening.

Totals of 4–8-week implantation periods are most often used to assess the degree of calcification of GA-fixed biomaterials in ectopic implantation models [36,37,38,39,40,41,42,43,44]. However, considering that SVD is characterized by long-term and slow changes [45], we additionally decided to use a longer implantation period (up to 24 weeks) in this study.

The animals from each group were randomly divided to be euthanized (carbon dioxide protocol) 8 and 24 weeks after the surgical procedure. 

To prevent autolysis, immediately after withdrawal, samples of implanted materials with surrounding tissues of the recipient bed were washed for 30 s with cold (14 °C) saline solution and fixed for 48 h in neutral buffered formalin (NBF) at a tissue-fixator volume ratio of 1:30.

### 2.8. Histological Analysis

After the termination of fixation, the fragments of samples were washed with distilled water (3 × 3 min) to remove excessive phosphates and placed for no less than 12 h in medium Optimum Cutting Temperature (O.C.T.) Compound Tissue Tek (Sakura, Tokyo, Japan). Cross slices of experimental samples (9 μm) were prepared by cryosectioning (MEV SLEE medical GmbH, Germany). The micrographs of stained histological samples were obtained on a Nikon Eclipse Ti-E microscope station (Nikon, Tokyo, Japan) and processed using the software NIS Elements AR4.13.05 (Build 933).

The qualitative content of acidic and neutral GAGS, as well as the number of residual aldehyde groups in the materials before and after treatment, were evaluated using Alcyan blue-PAS staining (the Mowry method in the Luna modification) [46]. 

The biocompatibility of these materials was assessed using H&E staining (Mayer’s Hematoxylin and Eosin Y), the number of cells that migrated into the matrix of materials (primarily fibroblast-like cells with elongated nuclei) was calculated, and the intensity of the inflammatory response was determined by the number of leucocytes (cells with rounded nuclei) migrating into the matrix. To assess the degree of cellular-resorptive processes, the preparations stained with the collagen/non-collagen structures differential staining (using Lillie’s trichrome method) were also evaluated [46]. 

The degree of calcification of the materials was assessed based on a study of histological preparations of explanted samples stained with different staining methods for calcium deposits using the Alizarin red S staining (by the McGee-Russell method [46]. 

The areas of collagen resorption and calcification were determined in the field of view (400 × 400 µm, *n* = 16) using the non-commercial ImageJ software (https://imagej.nih.gov (accessed on 16 March 2023)) and expressed as a percentage of the total area of the assessed area. 

### 2.9. Statistics

The results of in vitro studies are presented as mean ± standard deviation (M ± SD). Each in vitro experiment was performed at least five times (*n* ≥ 5). The statistical significance of the differences was determined using one-way ANOVA, followed by multiple Holm–Sidak comparisons (*p* < 0.05). The results of in vivo studies are presented as mean ± standard deviation (M ± SD). Each experiment was performed at least four times (*n* ≥ 4). The Mann–Whitney U-test was used to compare two independent samples; the differences between the groups were considered statistically significant (*p* < 0.05). The design of the experiment and related statistics were performed using SigmaPlot™ 14.0 (Systat Software Inc., San Jose, CA, USA). Plots were created using SigmaPlot™ 14.0.

For analyzing microscopic images, at least 10 fields of view per section were evaluated. Correlations between the studied variables were assessed using Spearman’s rank correlation coefficient. Statistical data processing was performed using Python 3 (ver. 3.10.10) in the development environment Spyder (v. 5.4.1) with libraries Pandas (v. 1.5.2), Numpy (v.1.24.2) and Scipy (v. 1.10.0). The graphical display of the obtained results was performed using Python 3 (version 3.10) with libraries Seaborn (v. 0.12.2) and Matplotlib (v. 3.7.0).

## 3. Results

### 3.1. Results of Damage Assessment

For all groups, the surface of *p.serosum* was studied in detail as the most important surface for evaluating the valve leaflets of BHVs experiencing the main hydrodynamic shock of blood flow. Considering that this surface should be as intact as possible in order to preserve the properties of thromboresistance, the surface was separately studied for the “exposure” of the collagen layer to be covered by the *p.serosum* lamina, as well as the presence or absence of areas of fibering and longitudinal micro-fractures. During the histological analysis of the matrix of materials after processing, the samples were examined for signs of structural disorganization of the ECM, as well as areas of micro-ruptures, fusion, and separation of collagen fibrils. The preservation of GAG in the matrix structure was separately investigated.

**Figure 1:**
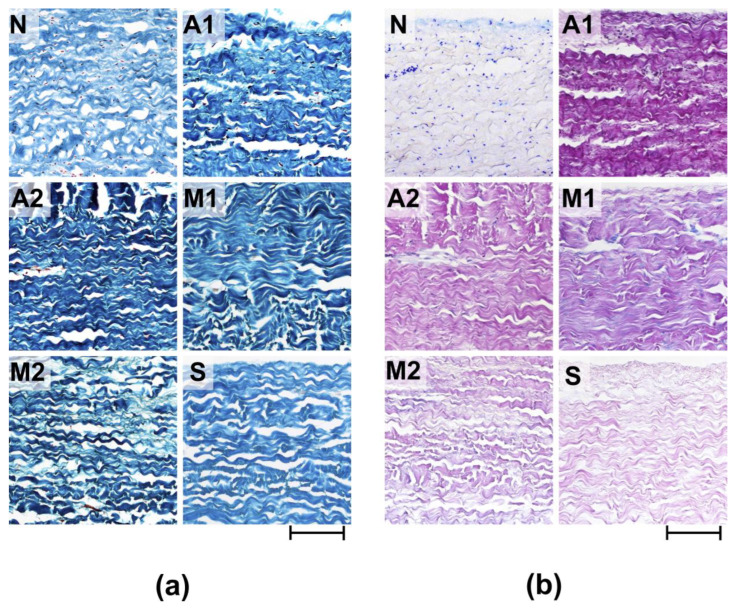
Histological analysis of experimental materials before (N) and after processing (preimplantation treatment). (**a**) Lillie’s trichrome stain (collagen is colored blue, non-collagen components are colored red, and cell nuclei are colored brown); (**b**) Alcian blue-PAS stain (acidic GAGs are stained blue, neutral GAGs are stained pink, cell nuclei—purple). Light microscopy; scale—100 µm.

Morphological analysis of the ECM materials after processing (preimplantation treatment) showed that the greatest degree of preservation of the ECM structure was observed in the samples of Group S (Figure 1a,b). The materials of Group M1 were characterized by pronounced damage to the collagen matrix, manifested in the fusion of collagen fibers in the middle part of the matrix and the rupture of the collagen fibers themselves, mainly on the side of *p.fibrosum*. In addition, in *p.fibrosum*, signs of transverse cracking of ECM materials were observed, which is a characteristic sign of loss of flexibility of collagen fibrils as a result of their dehydration. The most pronounced signs of longitudinal and transverse cracking of matrix fibers were observed in Group A2, both from the side of the *p.fibrosum* and from the side of the *p.serosum*. In addition, the matrix of materials of Groups A2 and A1 differed in the compacted structure typical of hyper-fixed biomaterials. In the M2 group, the most pronounced signs of reduction in the ECM were observed, which were thinning and partial straightening of collagen fibers, as well as their strong separation.

It should be noted that in Groups A1, A2, M1, and M2, the reduction in collagen fibers was combined with a change in their affinity for the dye, which manifested itself in varying degrees of dye impregnation (Fast Green FCF) of individual fibrillary bundles or the entire collagen matrix. In addition, a particularly high degree of dye saturation of the entire matrix was observed in Group A2, which indicates obvious dehydration and damage to the matrix of the materials in this group.

Fuchsinophilic saturation of collagen fibers was also characteristic of all the studied groups, which may be associated with both damage to the collagen matrix and GA concentration since the lowest degree of fuchsinophilia was observed for samples S and the highest for A1. In the samples of all studied groups after preimplantation treatment, acidic GAGs were not detected (Figure 1b).

**Figure 2:**
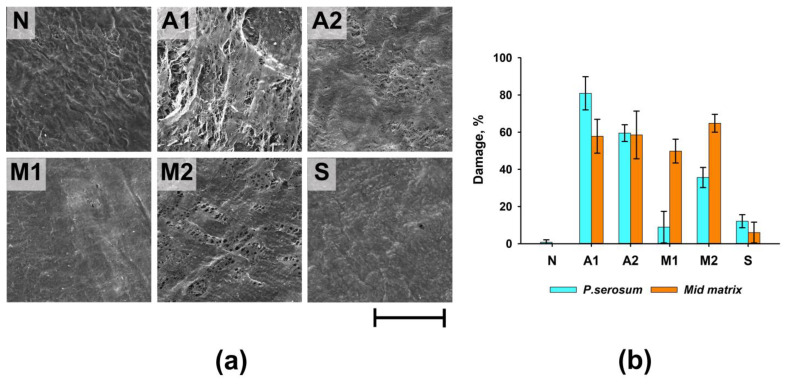
Results of morphological analysis of experimental samples before (N) and after processing. (**a**) SEM of *p.serosum* surface; scale—200 µm; (**b**) summary graph of the degree of damage to the surface of *p.serosum* and the medial matrix of ECM.

Group A1 had the greatest degree of damage to the surface of the materials (the area of damage to the *p.serosum* for this group reached 80.9 ± 8.9%), which manifested itself in partial detachment of the *p.serosum* from the underlying layer and strong collagen fibers separation of the underlying fibers (Figure 2a). In Groups A2 and M2, the surface damage was 35.6 ± 5.4 and 59.5 ± 4.5%, respectively. Damage to these groups was characterized by partial perforation of the upper layer of the surface and separation of the underlying fibers. In the M1 group, despite relatively medial matrix damage, serious damage to the *p.serosum* was not detected.

Thus, the maximum degree of damage to both the surface of the *p.serosum* and the medial part of the ECM was observed in Groups A1, A2, and M2, and the minimum degree of damage was observed in Group S (Figure 2b).

### 3.2. Results of Cytotoxicity and Cell Proliferation Assay

**Figure 3:**
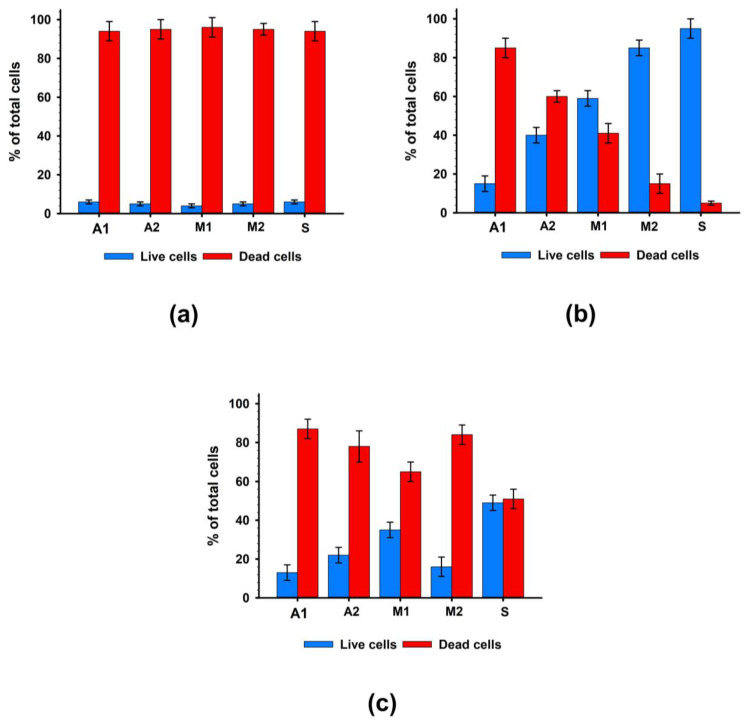
Results of cytotoxicity analysis after 96 h of cultivation with hMSC-AT cells. (**a**) Contact cytotoxicity with standard washing; (**b**) Contact cytotoxicity with prolonged washing; (**c**) Non-contact cytotoxicity.

An in vitro study of the contact toxicity of experimental materials during their cultivation with hMSC-AT cells showed that under standard washing conditions (2 h), all studied samples had a pronounced cytotoxic effect. After 96 h of cultivation, the number of living cells on the surface of the experimental biomaterials did not exceed 10.3 ± 6.2% (Figure 3a).

Additional washing (four days) significantly suppressed the cytotoxic effects of the biomaterials (Figure 3b). The materials of the M2 and S groups, in which cell survival was at least 80%, had the least cytotoxic effects on cells. In Groups A2 and M1, cell survival increased by four and six times, respectively. At the same time, in Group A1, cell survival after 96 h of cultivation remained low and did not exceed 15%. Thus, additional washing of samples simulating the leaching of chemical agents in the systemic bloodstream suppressed the contact cytotoxicity of biomaterial samples to the greatest extent for Groups M2 and S and to a lesser extent for Groups A2 and M1 but did not suppress the cytotoxicity in Group A1 samples.

A study of non-contact cytotoxicity showed that extracts from all groups of biomaterials had high toxicity to cells (Figure 3c), while the highest survival rate of cells with extracts was also observed in Group S.

**Figure 4:**
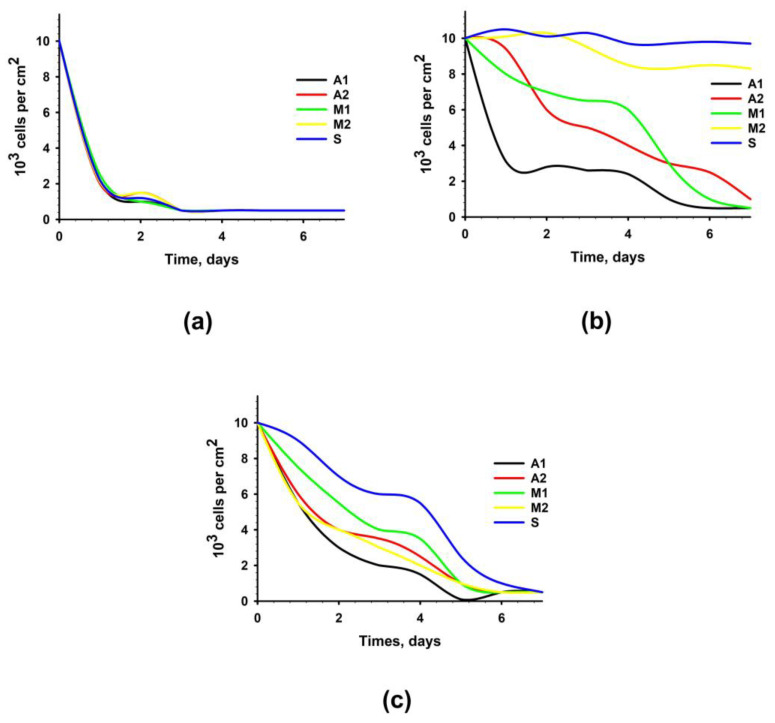
Proliferation activity of hMSC-AT cultivated for 7 days on the surface of experimental materials or with the addition of extracts from experimental materials. Dependence of the number of cells on the time of cultivation with standard (2 h) washing (**a**); with prolonged (four days) washing (**b**); with the addition of extracts from experimental materials (**c**).

When analyzing the proliferative activity and dynamics of the number of cell populations, it was found that the growth of cells cultured on samples from all experimental materials was completely absent. Simultaneously, a decrease in the number of cells was observed owing to the pronounced cytotoxic effect of the experimental samples (Figure 4a). Figure 4b shows the results of the analysis of the proliferative activity of cells cultured on samples of experimental materials after an additional 4-day washing. It was revealed that samples of materials from Groups A1, A2, and M1 not only prevented cell proliferation but also contributed to a decrease in their number due to the pronounced cytotoxic effect. In the case of samples from Groups M2 and S, no increase in the number of cells was recorded; however, no decrease in the number of cells was observed.

It was revealed that extracts from biomaterials of all groups not only prevented cell proliferation but also contributed to a decrease in their number due to the pronounced cytotoxic effect (Figure 4c).

The mitotic activity of hMSC-AT cultured on samples of all groups of biomaterials, both under the conditions of standard washing and additional washing, was completely absent. In the case of samples of experimental materials washed according to the standard procedure (2 h), the pronounced cytostatic effect was based on the direct toxic effect of the materials themselves. In the case of samples with additional 4-day washing in Groups A1, A2, and M1, the cytostatic effect was also mediated by the residual toxic effect of the materials themselves. In turn, the cytostatic effect of the biomaterial samples of Groups M2 and S may be due to the modification of the surface of the samples after processing and the weak residual toxic substances from the samples of materials.

### 3.3. Results of In Vivo Analisis

**Figure 5:**
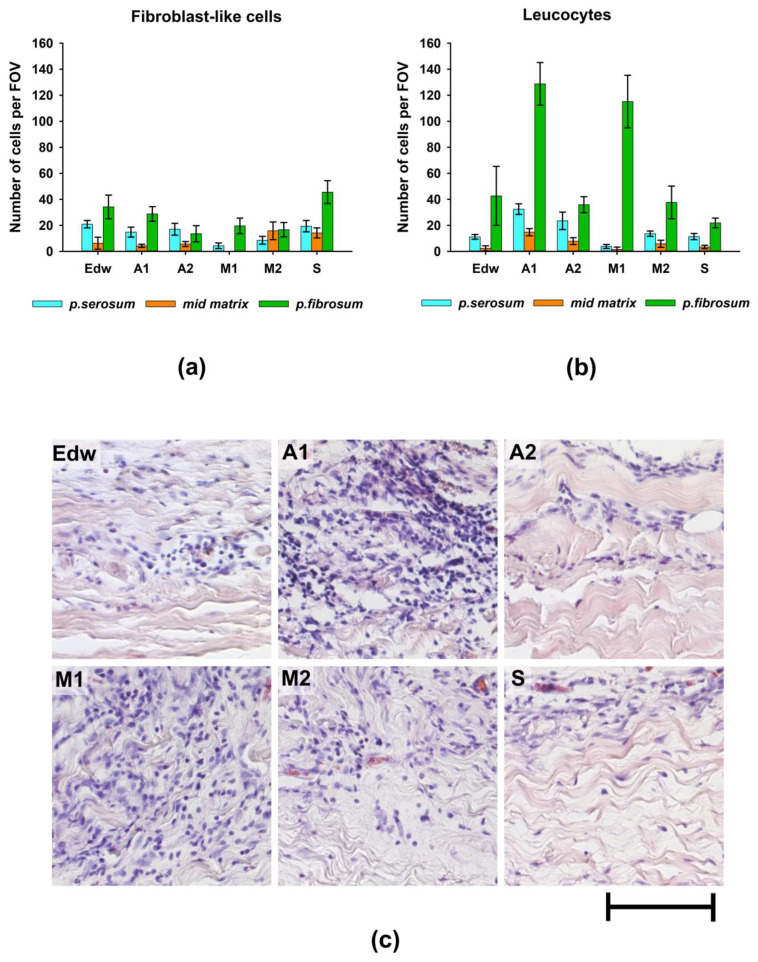
The results of the analysis of the biocompatibility of materials after 2 months of implantation in rats. Migration of (**a**) fibroblast-like cells and (**b**) leucocytes into various layers of the matrix of materials; (**c**) morphology of cells migrating from the *p.fibrosum*; hematoxylin and eosin staining (H&E, cell nuclei are colored blue, erythrocytes are colored red, and components of tissue are colored pink). Light microscopy; scale—50 µm.

Morphological analysis of the samples after 8 weeks of implantation showed that fibroblast-like cells migrated most actively to the medial matrix of the materials in Groups M2 and S (Figure 5a). At the same time, in the M1 group, cell migration to the medial matrix was almost completely absent, which may be due to the preservation of cytotoxicity of the materials in this group. In a comparative analysis of the results of cell migration in vivo and cell survival on the surface of materials in vitro, it was observed that fibroblast-like cells migrated most actively into the matrix of materials of those groups that had low cytotoxicity under conditions of their additional 4-day washing in culture medium.

Borderline migration of cells was observed mainly on the side of the *p.fibrosum*. An analysis of the morphology of cells that migrated to the border zones of the matrix found that in groups A1 and M1, most cells (>80%) had small and rounded nuclei, indicating the development of focal leucocytic invasion (Figure 5b,c). 

In Groups A2 and M2, the relative number of leucocytes was approximately 70%, whereas, in the Edw control group, the cell population was equally represented by leucocytes and fibroblast-like cells. In Group S, the number of migrated leucocytes did not exceed 32% (Figure 5). From the *p.serosum*, cells migrated in smaller numbers, mainly in groups where uniform cell migration into the matrix thickness was observed (all groups except M1).

**Figure 6:**
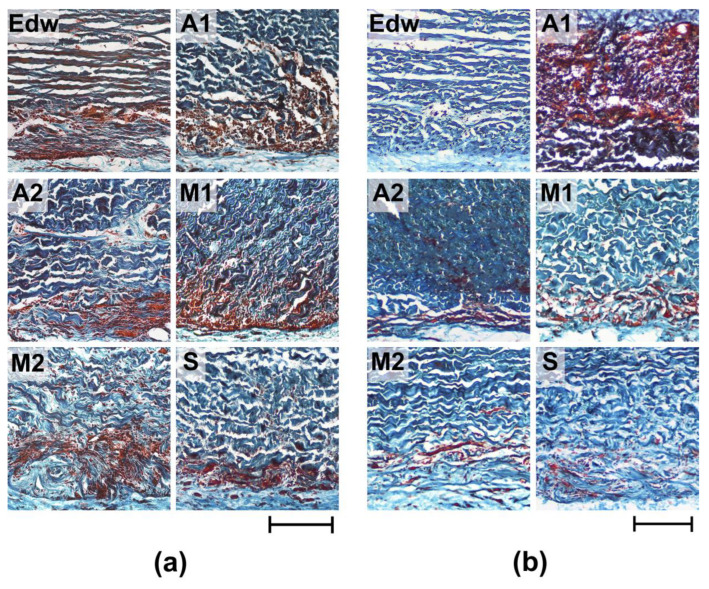
Histological analysis of resorption of implanted experimental materials after 8 (**a**) and 24 (**b**) weeks of implantation. Lillie’s trichrome staining (collagen is colored blue, muscle and other tissues are colored red, and cell nuclei are colored dark red/brown). Light microscopy; scale—200 µm.

Further analysis of the collagen matrix of the experimental materials showed that the areas of leukocyte invasion were co-localized with areas of resorption of VCM in all groups, which indicates the classical cellular nature of resorption (utilization) of damaged components of the extracellular matrix (Figure 6).

After 8 weeks of implantation, the most pronounced cellular matrix resorption (utilization) was detected in Groups A1 and M1; the resorption area was 38.12 ± 2.39 and 29.66 ± 1.09%, respectively (Figure 6a, Table 2). Simultaneously, the materials of all groups were characterized by maintaining the integrity of the medial thickness of the matrix. The exception was the M2 group, where boundary resorption had a focal character, and small areas of local resorption of the median matrix thickness were also observed. After 24 weeks of implantation, a decrease in the intensification of resorptive processes in the matrix was observed for materials of all groups; the resorption area did not exceed 15% (Figure 6b, Table 2). The exception was the materials of Group A1, where, within 6 months of implantation, the resorption area had increased to almost 50%. Only Group S showed no signs of active cell-resorptive processes, as evidenced by the absence of signs of borderline leucocytic invasion, as well as resorption of the collagen matrix at all implantation periods.

**Figure 7:**
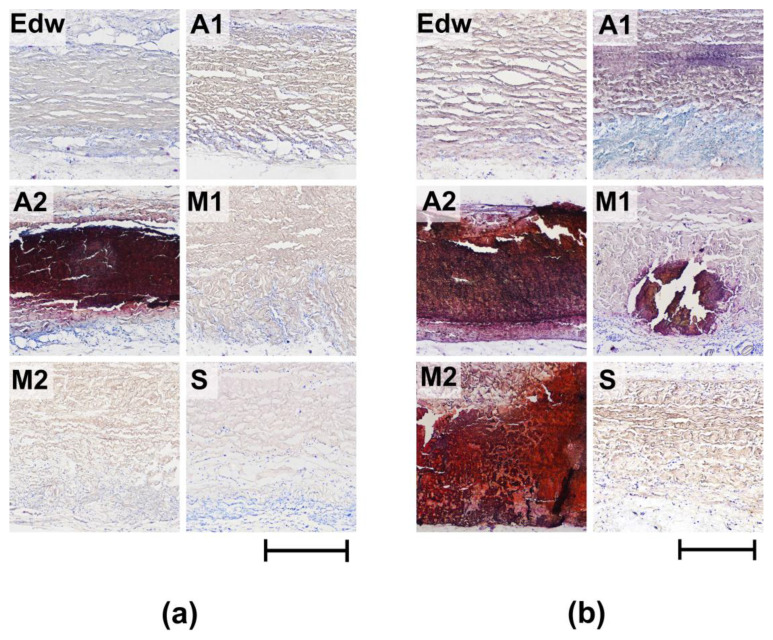
Histological analysis of calcification of implanted experimental materials after 8 (**a**) and 24 (**b**) weeks of implantation. Alizarin red S Staining with a toluidine blue dyeing (calcium deposits are colored orange-red and dark-red, cell nuclei are colored blue, and mast cells are colored purple). Light microscopy; scale—500 µm.

In Group A2 samples, pathological calcification was observed 8 weeks after implantation. Mineralized samples from this group were characterized by low rates of resorption of the collagenic matrix (Table 2, Figure 7a). Groups M1 and M2 underwent mineralization only at later implantation periods (24 weeks), accompanied by a significant decrease in the intensification of cell-resorptive processes (Figure 7b). At the same time, in the M1 group, calcification intensity was relatively low, which may be due to the low degree of repopulation of the medial matrix by recipient cells. In Group A1, in which there were no signs of aseptic calcification, the maximum degree of cellular resorption was observed with the migration of a large number of resorbing cells, even at late periods of 24 weeks.

Group S and the control group Edw showed no signs of calcification during any of the implantation periods.

### 3.4. Results of Correlation Assay

To identify the dependence of the calcification of materials on previously identified parameters (cytotoxicity, matrix damage, cell migration, and the degree of resorption), a correlation analysis was performed. Group A1 was excluded from this analysis because in vivo studies showed that the materials of this group underwent intensive cellular resorption, bypassing the calcification stage. The power (strength) of the correlation was determined, as shown in Table 3.

**Figure 8:**
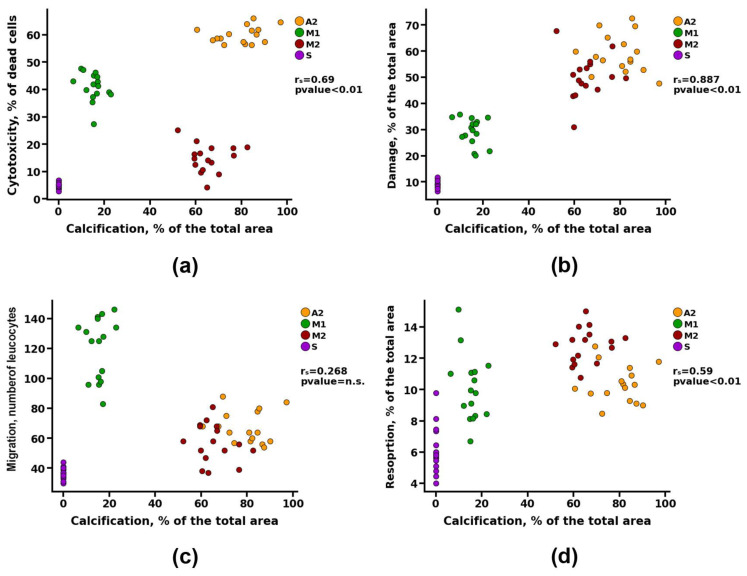
Spearman correlation analysis between the degree of calcification and (**a**) value of cytotoxicity, (**b**) matrix damage, (**c**) cell migration, and (**d**) resorption.

**Figure 9:**
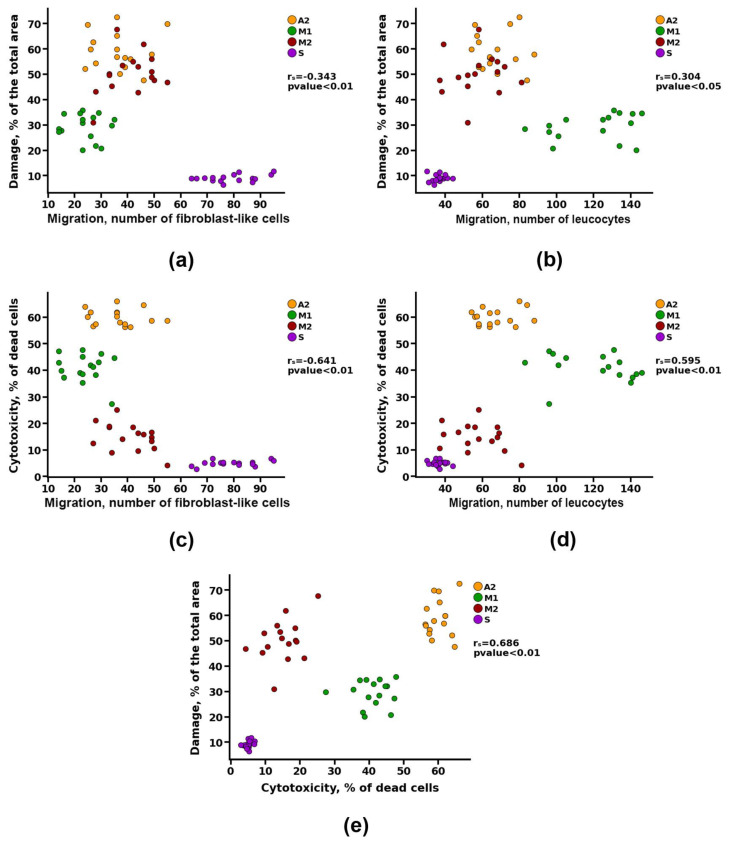
Spearman correlation analysis between cell migration and (**a**,**b**) matrix damage and (**c**,**d**) value of cytotoxicity, and between (**e**) matrix damage and value of cytotoxicity.

Correlation analysis revealed a noticeable correlation between the calcification of materials and parameters such as cytotoxicity and resorption (r_s_ > 0.5). However, a detailed study of these data presented in Figure 8a shows that materials with both high and low cytotoxicity can undergo intense calcification (Groups A2 and M2, respectively). Probably, there is a contribution from the third parameter, which can affect both the survival of cells on materials and the degree of their calcification. Such a parameter may be the degree of damage, for which the correlation with the calcification index was defined as high (r_s_ = 0.88) (Figure 8b) and with the cytotoxicity index—noticeable (r_s_ = 0.69) (Figure 9e).

At the same time, no correlation was found between the migration of leukocytes into the matrix of the materials and its calcification (Figure 8c). However, there was a noticeable correlation between the indicators of cell migration and the degree of cytotoxicity (r_s_ > 0.5) and a moderate correlation between the indicators of cell migration and damage to the matrix of materials (r_s_ > 0.3) (Figure 9).

Apparently, damage to materials during preimplantation treatment leads to a decrease in cell survival, which may be due to both modification of the surface in contact with cells and the increased leaching of cytotoxic substances from the materials. The damaged material also attracts more leukocytes to the implantation area and reduces the migration of fibroblast-like cells, which is caused by two factors: the death of the recipient’s cells in contact with toxic material and the initiation of cell-resorptive processes in response to the damaged collagen matrix. Together, these effects increase the likelihood of the development of intensive aseptic calcification of GA-fixed materials.

Thus, general damage to the structure of materials by more than 40% leads to the development of intense calcification, whereas slightly damaged materials (20–40%) undergo less intense calcification. In addition, as shown earlier for the A1 group materials, damage to materials by more than 70% initiates the development of their rapid cellular resorption (utilization).

A low degree of damage to the VCM significantly reduced the immunogenic properties of materials (cytotoxicity and leukocyte invasion), increased the potential of materials for re-endothelization, and contributed to its resistance to calcinosis, which was shown for group S.

## 4. Discussion

The proposed mechanisms of calcification initiation include two possible pathways: (a) the release of calcium ions from dying cells due to the cytotoxic effect of GA [47,48,49] and (b) the development of an immune response and activation of macrophages that release MMPs and other proteases that promote the opening of calcium-binding sites on damaged collagen [6,50], the release of calcium-binding proteins on the background of proteolytic cleavage of mineralization inhibitors (osteopontin, MGP, etc.) [51].

Our study confirmed the dependence of aseptic calcification on the immune response and the launch of cellular resorptive processes. The correlation between the degree of damage to materials after processing and the degree of calcification indicates that the immune response and subsequent calcification, in this case, developed in response to damage patterns of the implanted collagen matrix by the mechanism of the DAMP-associated immune response. Fragmented components of the extracellular matrix, such as GAGs or fibronectin, are among the most well-known DAMPs that are released due to rigid decellularization [52,53]. The recognition of DAMPs by various non-immune cells and innate immune cells leads to the release of pro-inflammatory cytokines, attracting more immune cells to the implantation area and polarizing macrophages into the M1 phenotype [54,55,56,57] and MMP production [55,58]. Damage and denaturation of ECM components using harsh decellularization methods can also expose some hidden spiral and terminal antigenic sites of collagen, which trigger the production of antibodies [59] and the subsequent immune response. Some latent laminin motifs of *p.serosum* induce chemotaxis of macrophages and neutrophils and increase MMP activity [60]. Furthermore, immune cells and MMPs can destroy the ECM and expose the three-peptide motifs of PGP (Pro-Gly-Pro), which trigger the CXCR2 receptors of neutrophils, control their chemotaxis, and initiate interactions with cells of adaptive immunity [22,61]. This is how the formation of a vicious circle occurs, leading to the establishment of chronic inflammation and the utilization of biological material that was shown for Groups A1, A2, M1, and M2.

Thus, violation of the ECM integrity of BHVs during preimplantation treatment can directly increase their immunogenicity. Simultaneously, it can be assumed that a high degree of matrix damage leads to direct proteolytic destruction of the BHVs matrix, bypassing the intermediate stage of calcification, which was demonstrated in Group A1, which had the greatest degree of damage, pronounced leukocyte invasion, and lack of calcification against the background of active cellular proteolytic resorption.

In this study, the areas of the matrix of experimental materials that underwent active degradation were not mineralized, whereas the zones of calcification nucleation were observed exactly where active cellular migration was accompanied by a decrease in the intensification of the processes of ECM resorption. Thus, it was shown that in the case of free migration of cells into the entire thickness of the matrix, damaged areas that are not amenable to direct cellular and proteolytic degradation undergo intensive mineralization after eight weeks of implantation, as was observed for Group A2. Conversely, in the M2 group, which was also actively repopularized by the recipient cells in the early stages of implantation, there were no signs of calcification, but there were signs of active resorption of the border regions and even foci of resorption of the matrix thickness itself. However, at the later implantation dates (24 weeks), part of the extracellular fibrillar matrix that was apparently relatively well preserved and could not be directly disposed of due to the proteolytic activity of cells underwent an intermediate phase of calcification, which is essentially a form of disposal of a foreign body. Finally, the A1 group materials, where degradation and cellular resorption of the matrix of materials were observed even after six months of implantation, showed no signs of calcification since the severely damaged matrix could be resorbed directly by recipient cells and intercellular fluid proteases. Thus, the observed cell-resorptive processes preceding calcinosis (especially the M2 group) may indicate the utilization nature of this process; that is, calcification, in this case, maybe one of the stages of the process of utilization of the implanted matrix, perceived by the body as a foreign body that is not resorbed through proteolysis.

Several researchers have assumed that GA can initiate calcification. There are theories that both residual aldehyde groups and changes in the structure and surface charge of collagen molecules can contribute to calcification [41,62]. At the same time, the results of in vivo studies on materials may report data contradicting the above theories. Thus, some studies have shown that after two months of implantation, the average calcium content in pericardial tissues fixed at 0.6% GA was significantly lower than that in pericardial tissues fixed at 0.4% GA [63]. Thus, some researchers suggest that the use of GA at higher concentrations (0.5–0.6%) is preferable because such materials have superior mechanical characteristics and are more resistant to enzymatic degradation and post-implantation calcinosis. In this study, no correlations were found between the cytotoxicity of the materials and the intensity of calcification, which casts doubt on the assumption that the cytotoxicity of GA can contribute to calcification. However, as was shown for the M1 group, cytotoxicity of GA can hinder the migration of recipient cells into the matrix of materials and, thus, slow down the development of cellular resorptive processes and only delay the development of calcification. At the same time, such an effect does not seem to depend on the concentration of GA but on the rate of release/leaching of the toxic agent from the material, which also correlates with its structural damage; that is, the lower the degree of damage to the surface, the slower the GA is extracted. The use of long implantation periods (24 weeks) revealed the true tendency of a number of materials to delay mineralization, which correlates well with clinical data.

It should be noted that the use of fixing agents (GA and formaldehyde) as a component of storage solutions contributes to the visual restoration of the macrostructure of the *p.serosum* of the materials, which was observed for materials of the M1 group in comparison with M2. Such a “restoring” effect of storage solutions can lead to an incorrect assessment of the suitability of the finished material since the results of histological analysis of the collagen matrix showed the presence of a wide range of different types of damage, even in the M1 group.

At the same time, by analyzing the results obtained, we can safely assume that the use of gentle methods of preimplantation processing of materials that ensure maximum preservation of the intactness of the ECM makes it possible to radically increase the resistance of pericardial materials to calcinosis, as shown for Group S materials.

## 5. Conclusions

Thus, the use of aggressive methods for processing biomaterials (SDS, GA, and alcohols in high concentrations, especially isopropyl) leads to damage and changes in the structure of the matrix and opens up patterns of ECM damage, initiating an immune response aimed at the utilization of damaged matrix components by recipient cells. If direct proteolytic utilization by cells of individual sections of the GA-fixed matrix (insufficiently damaged) is impossible, an intermediate phase of calcification is initiated, preceding the intensive utilization of the mineralized fibrillar matrix by osteoclast-like cells. With this form of damage to the extracellular matrix of BHVs, signs of calcification appear mainly by late implantation (at least 20–24 weeks), after which it is necessary to use long implantation periods in laboratory animals to assess the true tendency of biomaterials to undergo calcification. A high degree of cytotoxicity of materials, which is not related to the concentration of GA used but is associated with the degree of damage to the surface of the biomaterial and the rate of release of toxic substances, can delay calcification, blocking access to the matrix of materials for cells, and thus inhibiting the development of calcification. At the same time, despite such a delay, calcification of damaged but chemically stabilized materials will still occur and will most likely occur in an avalanche, as is often observed in clinical practice.

In summary, we can safely assume that the use of sparing methods for preimplantation processing of materials that ensure maximum preservation of the intactness of the ECM of the pericardium allows us to radically increase the resistance of biomaterials to aseptic calcinosis, as was shown for group S materials.

## Figures and Tables

**Table 1:** Processing of biomaterials.

Group	Decellularization	Cross-Linking	Washing	Preservation
A1	1% SDS	0.625% GA, 4% FD	0.9% NaCl	GA, EtOH
A2	1% SDS	0.5% GA	70% IPA, 90% EtOH	FD, GA, EtOH
M1	Triton X-100, SDC	0.2% GA	35% IPA	GA, EtOH
M2	Triton X-100, SDC	0.2% GA	35% IPA	DN, EtOH
S	Triton X-100, SDC	0.2% GA	30% EtOH	DN, EtOH

SDS—Sodium Dodecyl Sulfate; SDC—Sodium Deoxycholate; GA—Glutaraldehyde; FD—Formaldehyde; IPA—Isopropyl Alcohol; EtOH—Ethanol, Ethyl Alcohol; DN—Dioxidine.

**Table 2:** Comparative analysis of resorption and calcification of experimental materials 2 and 6 months after implantation.

Group	8 Weeks		24 Weeks	
	Resorption, %	Calcification, %	Resorption, %	Calcification, %
Edw	11.4 ± 1.21	0	2.6 ± 1.05	0
A1	38.12 ± 2.39	0	49.22 ± 6.93	0
A2	15.86 ± 3.45	33.8 ± 5	10.36 ± 1.15	79.7 ± 9.3
M1	29.66 ± 1.09	0	10.08 ± 2.05	15.3 ± 4
M2	20.36 ± 1.33	0	12.79 ± 1.1	65.5 ± 7.5
G	10.46 ± 1.88	0	6.12 ± 1.41	0

**Table 3:** Interpretation of Spearman’s rank correlation coefficient.

Coefficient Range (Absolute Value), r_s_	Interpretation
<0.3	Low correlation
0.3–0.5	Moderate correlation
0.5–0.7	Noticeable correlation
0.7–0.9	High correlation
>0.9	Very high correlation

## Data Availability

The data presented in this study are contained within this article and online supplemental data.
